# Depletion of Mitochondrial Cyclophilin D in Endothelial and Smooth Muscle Cells Attenuates Vascular Dysfunction and Hypertension

**DOI:** 10.1093/function/zqaf006

**Published:** 2025-02-07

**Authors:** Anna Dikalova, Mingfang Ao, Louise Lantier, Sergey Gutor, Sergey Dikalov

**Affiliations:** Vanderbilt University Medical Center, Nashville, TN, USA; Vanderbilt University Medical Center, Nashville, TN, USA; Vanderbilt University, Nashville, TN, USA; University of Michigan, Ann Arbor, MI, USA; Vanderbilt University Medical Center, Nashville, TN, USA

**Keywords:** hypertension, vascular dysfunction, mitochondria, cyclophilin D, superoxide, glycolysis

## Abstract

Hypertension is a major risk factor of cardiovascular disease affecting nearly half of adult population. It is associated with mitochondrial dysfunction and understanding these mechanisms is important to develop new therapies. Cyclophilin D (CypD) promotes mitochondrial swelling and dysfunction. The objective of this study is to test if CypD depletion attenuates vascular dysfunction and hypertension using endothelial and smooth muscle-specific CypD knockout mice in angiotensin II model of vascular dysfunction and hypertension. Our results show that depletion of endothelial CypD prevents angiotensin II-induced impairment of endothelial-dependent vasorelaxation, preserves endothelial nitric oxide and mitochondrial respiration, attenuates hypertension, vascular oxidative stress and vascular metabolic glycolytic-switch. Depletion of smooth muscle CypD slightly reduces angiotensin II-induced hypertension, protects vascular nitric oxide and vasorelaxation, decreases vascular superoxide, diminishes angiotensin II-induced vascular glycolysis, hypertrophy and fibrosis. These data suggest “metabolic” and “redox” crosstalk between endothelial and smooth muscle cells. Endothelial CypD depletion reduces not only endothelial glycolysis but also attenuates smooth muscle cell glycolytic switch. Smooth muscle CypD depletion reduced not only smooth muscle glycolysis, but it also attenuated endothelial glycolysis. Vascular oxidative stress was inhibited both in EcCypDKO and SmcCypDKO mice, therefore, cell-specific CypD depletion had “global” antioxidant effect in vasculature. Our results support a novel function of mitochondrial CypD in regulation of superoxide and metabolism in vascular smooth muscle and endothelial cells which affect endothelial barrier and smooth muscle vascular functions. We suggest that blocking vascular CypD reduces vascular oxidative stress, improves vascular metabolism and vascular function which may be beneficial in cardiovascular disease.

## Graphical Abstract

**Graphical Abstract:**
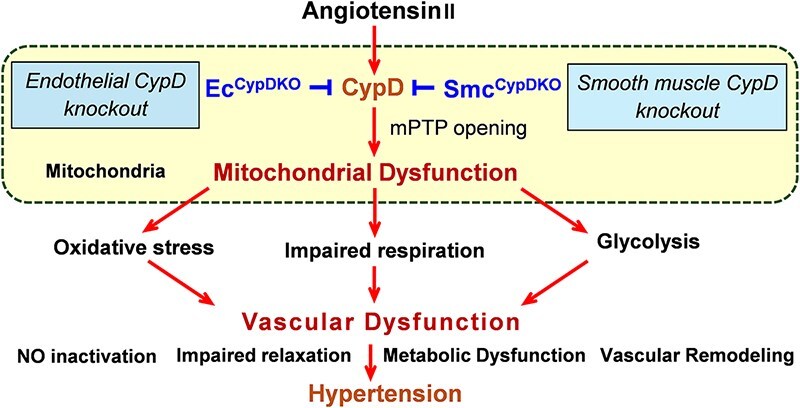
Our overarching hypothesis is that cell-specific cyclophilin D (CypD) depletion in endothelial and smooth muscle cells attenuates vascular dysfunction and hypertension. To test this hypothesis, we have developed tamoxifeninducible endothelial-specific and tamoxifen-inducible smooth muscle cell–specific CypD knockout mice. Our studies of angiotensin II–induced hypertension, endothelial-dependent relaxation, endothelial barrier permeability, vascular superoxide production, vascular mitochondrial respiration and glycolysis, aortic hypertrophy, and fibrosis support the novel role of CypD in the regulation of vascular oxidative stress, metabolism, and cellular functions.

## Introduction

Hypertension is a multifactorial disorder in response to alterations in neural, endocrine, and immune systems, hemodynamics, genetic factors, and maladaptive changes in the vasculature.^1^ There is an unmet need for the treatment of hypertension since only 25% of hypertensive patients have their blood pressure under control potentially due to the mechanisms that are not affected by current treatments. Vascular dysfunction plays a critical role in the development of hypertension and hypertensive end-organ damage.^2^ It is associated with mitochondrial dysfunction^3^; however, molecular mechanisms of mitochondrial dysfunction and its causative role in hypertension are not clear. Interestingly, mitochondria are a common target of cardiovascular risk factors such as age, diet, cigarette smoking, sedentary lifestyle, and metabolic conditions.^4^ Mitochondria are critical in cell metabolism and function, and, therefore, it is conceivable that mitochondrial impairment can drive vascular dysfunction and contribute to development of hypertension.

Previous studies have identified multiple mitochondrial pathways linked to hypertension including apoptosis, calcium homeostasis, mitochondrial metabolism, and oxidative stress.^5^ Several enzymes can serve as a “master” regulator of mitochondrial function such as peroxisome proliferator–activated receptor γ coactivator 1α, nuclear respiratory factor 1, and deacetylase sirtuin 3.^6–8^ There is a new potential candidate, cyclophilin D (CypD).^9^ CypD is an important mitochondrial chaperone protein; however, its specific mechanisms of action remain unclear. CypD can act as a peptidyl-prolyl, cis-trans isomerase involved in mitochondrial protein folding. Indeed, CypD promotes the assembly of mitochondrial ATP synthase into synthasome supercomplex.^10^ Meanwhile, CypD interaction with ATP synthase β-subunit F1 is likely responsible for calcium-sensitive mitochondrial permeability transition pore (mPTP) opening,^11^ which is linked to mitochondrial swelling, cellular apoptosis, and necrosis.^12^^,^^13^ Potential role of CypD in redox signaling includes ischemic preconditioning,^14^ regulated mitochondria superoxide production,^15^ pathogenic superoxide overproduction, and endothelial dysfunction.^16^ These data suggest that CypD is implicated in both mitochondrial homeostasis and mitochondrial dysfunction. These seamlessly contradictory functions of CypD can be potentially explained by post-translational modifications of CypD such as S-glutathionylation^16^ and acetylation,^17^ which can lead to a “gain of function” and switch from homeostatic to pathogenic function. Meanwhile, targeting CypD is hindered by the lack of specificity of known CypD blockers.^18^ Off-target effects of cyclosporine A paradoxically lead to increased sympathetic outflow, endothelin production, vasoconstriction, and hypertension, which are likely associated with calcineurin inhibition.^19^ CypD blocker sanglifehrin A^20^ attenuates hypertension,^16^ but it has off-target immunosuppressive effect and cannot be used orally.^21^ It is, therefore, unclear whether CypD inhibition is protective or detrimental to vascular functions due to nonspecific blockade of both homeostatic and pathogenic CypD functions.

We hypothesized that cell-specific CypD depletion in endothelial and smooth muscle cells attenuates vascular dysfunction and hypertension. To test this hypothesis, we have developed tamoxifen-inducible endothelial-specific CypD knockout mice (Ec^CypDKO^) and tamoxifen-inducible smooth muscle cell–specific CypD knockout mice (Smc^CypDKO^). We performed studies of angiotensin II (Ang II)–induced hypertension, endothelial-dependent relaxation, endothelial barrier permeability, vascular superoxide production, vascular mitochondrial respiration and glycolysis, aortic hypertrophy, and fibrosis. Our data support a multifaceted role of CypD in the regulation of vascular oxidative stress, metabolism, and cellular functions.

## Materials and Methods

The authors declare that all supporting data are available within the article and its online supplementary files. All methods have corresponding literature references. Additional protocol information is available from the corresponding author upon reasonable request.

### Reagents

Cellular superoxide probe was supplied by Invitrogen (Grand Island, NY). CypD (ab110324) antibodies were from Abcam. SOD2 (sc30080) antibodies were from Santa Cruz Biotechnology. Secondary antibodies conjugated with horseradish peroxidase were purchased from Amersham (anti-Rabbit IgG NA934V or anti-Mouse IgG NA931V). All other reagents were obtained from Sigma (St Louis, MO).

### Animal Experiments

To test the pathophysiological role of vascular CypD, we developed new endothelial cell–specific tamoxifen-inducible CypD knockout mice (Ec^CypDKO^) by crossing CypD^flox/flox^ mice (Jacson Labs, stock # 005737) with mice transgenic for Cre recombinase driven by a tamoxifen-inducible endothelium VeCad promoter provided by Dr Rolf Adam (University of Münster, Germany).^22^ To delete CypD in vascular smooth muscle cells, we crossed CypD^lox/lox^ mice with mice transgenic for Cre recombinase driven by tamoxifen-inducible Cre in the vascular smooth muscle (inducible SMMHC Cre) provided by Prof. Stefan Offermanns (University of Heidelberg, Germany).^23^ To induce CypD deletion, mice double positive for floxed sequence and Cre were injected with a low dose of tamoxifen (2 mg/30 g) for 5 days starting at 3 months of age, which have minimal transient cardiovascular effect.^24^ Wild-type Cre-negative littermates were also treated with tamoxifen. Two weeks later, hypertension was induced by Ang II (0.7 mg/kg/day, 14 days) in 4- to 5-month-old male mice.^25^ Blood pressure was monitored by the telemetry and tail-cuff measurements as previously described.^26^^,^^27^ Mice were housed in a temperature-controlled environment with 12-h light/dark cycles where they received food and water ad libitum. The Vanderbilt Institutional Animal Care and Use Committee approved the procedures (Protocol M1700207). Simple randomization was used to select animals for sham or Ang II for equal chance of being allocated to treatment groups.

### Superoxide Measurements Using HPLC

Mouse aortic segments were loaded with superoxide probe dihydroethidium (DHE) (50 µm) in Krebs/HEPES buffer for 30-min incubation in a tissue culture incubator at 37°C. Then, the tissue was placed in methanol (300 µL) and homogenized with a glass pestle. The homogenate was passed through a 0.22 μm filter and filtrates were analyzed by .^28^ The superoxide-specific product mito-2-hydroxyethidium was detected using a C-18 reverse-phase column (Nucleosil 250 to 4.5 mm) and a mobile phase containing 0.1% trifluoroacetic acid and an acetonitrile gradient (from 37% to 47%) at a flow rate of 0.5 mL/min. DHE superoxide–specific product 2-hydroxyethidium was detected using a C-18 reverse-phase column (Nucleosil 250 to 4.5 mm) as described previously.^29^

### Nitric Oxide Measurements by Electron Spin Resonance

Endothelial nitric oxide was quantified by electron spin resonance (ESR) and colloid spin trap Fe(DETC)_2_.^30^ All ESR samples were placed in quartz Dewar (Corning, New York, NY) filled with liquid nitrogen. ESR spectra were recorded using an EMX ESR spectrometer (Bruker Biospin Corp., Billerica, MA) and a super-high Q microwave cavity. The ESR settings were as follows: field sweep, 160 gauss; microwave frequency, 9.42 GHz; microwave power, 10 mW; modulation amplitude, 3 gauss; scan time, 150 ms; time constant, 5.2 s; and receiver gain, 60 dB (*n* = 4 scans).

### Western Blotting

Mouse aortas were homogenized in RIPA lysis buffer (Sigma, R0278) with 2.0 mmol/L sodium orthovanadate (Na_3_VO_4_), 1.0 mm fluoride phenylmethylsulfonyl containing inhibitors of proteolytic enzymes: 10 μg/mL aprotinin, 10 μg/mL leupeptin, and 10 μg/mL pepstatin (Sigma-Aldrich). The concentration of protein in lysates was determined using the protein assay kit (Bio-Rad). Protein (20 μg) separation was carried out in polyacrylamide gels (4%-12%) and transferred to PVDF membrane (Bio-Rad) at 4°C. Nonspecific binding sites on the membrane were blocked with 5% skim milk or 3% BSA in Tris-buffered saline solution with Tween for 1 h at room temperature and then membranes were incubated with anti-CypD (Abcam, ab110324, 1:1000) or anti-SOD2 (sc30080, 1:1000) overnight at 4°C. The next day, membranes were incubated with secondary antibodies conjugated with horseradish peroxidase. Pierce ECL Western Blotting Substrate (Thermo Fisher Scientific) was used for chemiluminescence-based detection. Final scans were performed using X-ray films or Azure c500 Western Blot Imaging System (Azure Biosystem). Densitometric analyses were performed using ImageJ software or ImageStudio software (LI-COR). Data were normalized by GAPDH levels.

### Vasodilatation Study

Isometric tension studies were performed on 2-mm mouse aortic rings dissected free of perivascular fat. Studies were performed in a horizontal wire myograph (DMT, Aarhus, Denmark, models 610M and 620M) containing physiological salt solution with the composition of 118 mm NaCl, 4.7 mm KCl, 1.2 mm MgSO_4_, 1.2 mm KH_2_PO_4_, 25 mm NaHCO_3_, 11 mm glucose, and 1.8 mm CaCl_2_. The isometric tone of each vessel was recorded using LabChart Pro v7.3.7 (AD Instruments, Australia). The aortic rings and mesenteric arteries were equilibrated over 2 h by heating and stretching the vessels to an optimal baseline tension before contracting them with 60 mm KCl physiological saline solution. Endothelial-dependent and endothelial-independent vascular relaxation were tested after preconstriction with 1 µm phenylephrine. Once the vessels reached a steady-state contraction, increasing concentrations of acetylcholine or sodium nitroprusside were administered, and the response to each concentration of drug was recorded.

### Endothelium-denuded Aorta

Mice were euthanized with carbon dioxide and aortas were gently excised, placed in cold buffer. Fat and connective tissues were removed carefully. The aorta was opened longitudinally with scissors. This preparation preserves intact endothelial cell function as measured by endothelial nitric oxide. The endothelium was mechanically removed by gently rubbing the intimal surface.^31^

### Lactate Measurements

Vascular glycolysis rate was measured in aortas isolated from sham and Ang II–infused mice and the descending part of aorta (6 mg) was placed in (glucose 1 g/L) tissue culture for 24 h.^17^ Glucose uptake and lactate production were tested in dulbecco's modified eagle medium (DMEM) aliquots using the hexokinase/glucose-6-phosphate dehydrogenase enzymatic assay and the l-lactate colorimetric assay (abcam kit ab65331).

### High-resolution Respirometry on Isolated Aortas

Measurements of respiration on isolated aortas were performed as previously described.^32^ Aortas were isolated from sham and Ang II–infused mice and sections (∼6-10 mg) were placed in ice-cold BIOPS buffer containing CaK_2_EGTA (2.77 mm), K_2_EGTA (7.23 mm), Na_2_ATP (5.77 mm), MgCl_2_·6H_2_O (6.56 mm), Na_2_phosphocreatine (15 mm), imidazole (20 mm), taurine (20 mm), and K-MES (50 mm), pH 7.1. Aortas were then permeabilized in ice-cold BIOPS containing 50 µg/mL saponin for 30 min, then washed in respiration buffer Mir05 containing sucrose (110 mm), K-lactobionate (60 mm), HEPES (20 mm), taurine (20 mm), K_2_HPO_4_ (10 mm), MgCl_2_·6H_2_O (3 mm), EGTA (0.5 mm), and fatty acid–free BSA (0.1%), pH 7.1. Aortas remained in fresh ice-cold Mir05 under gentle agitation until respiration analysis. Mitochondrial respiration was measured using the oxygraph O2k (Oroboros) with continuously stirred chambers maintained at 37°C. The oxygen consumption related to Krebs cycle/complex I–linked substrates, NADH donors (2 mm malate + 5 mm pyruvate), was measured in the presence of 2 mm ADP. The uncoupler CCCP was added after ADP to assess maximal uncoupled respiration (1 μm). Results are presented as oxygen flux normalized to wet tissue weight (pmol/s/mg weight).

### Statistics

The normality of continuous variable distribution was examined with the Shapiro-Wilk test. Normally distributed data are expressed as mean ± SD and nonnormally distributed data are expressed as median (Q25-Q75). Comparisons of the normally distributed continuous variables were assessed by the 2-way analysis of variance (ANOVA) followed by a Tukey post hoc test. Otherwise, we used appropriate nonparametric tests (Mann-Whitney and Kruskal-Wallis for 2 and >2 group comparisons, respectively). For telemetry blood pressure measurements over time and aortic relaxation, a 2-way ANOVA with repeated measures was employed. All statistical analyses were done using GraphPad Prism 10. *P* values < .05 were considered significant.

## Results

### Endothelial CypD Depletion Prevents Endothelial Dysfunction and Reduces Hypertension

**Figure 1.**
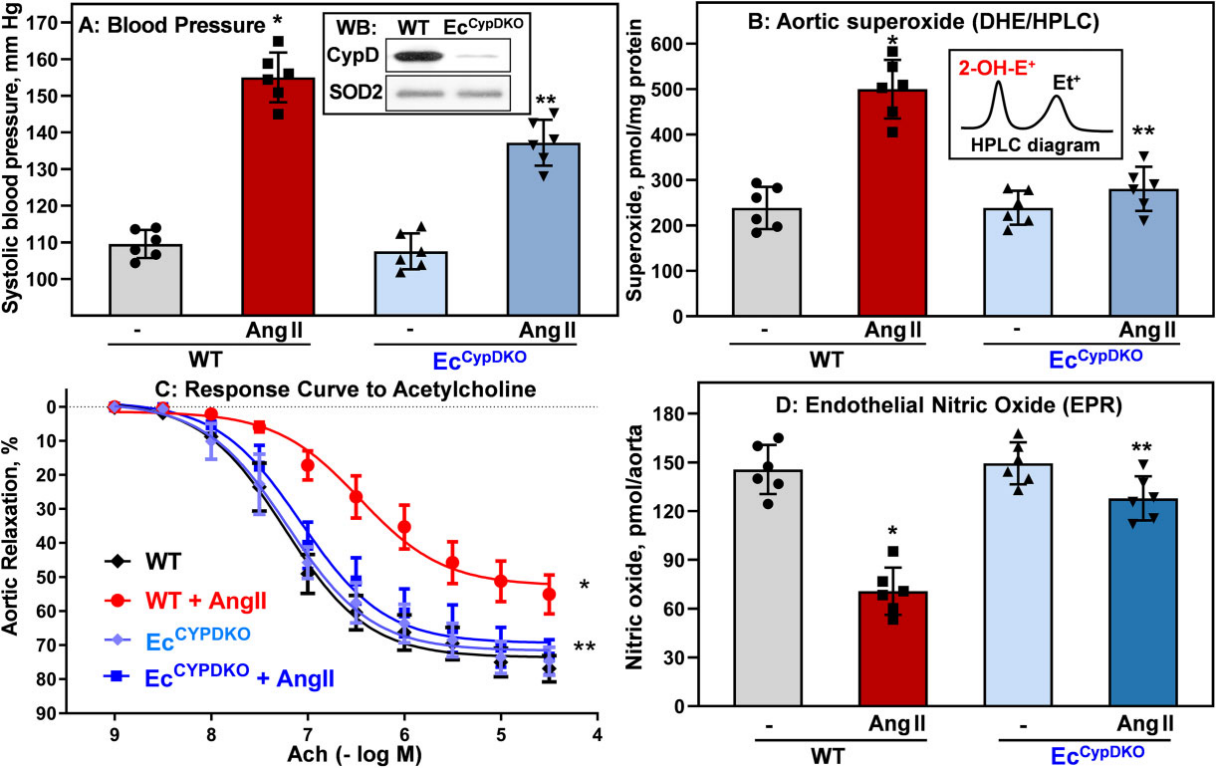
Systolic blood pressure, vascular oxidative stress, and endothelial function in sham and angiotensin II (Ang II)–infused Ec^CypDKO^ and wild-type (WT) male mice. (A) Systolic blood pressure after 14 days of angiotensin II infusion was measured by telemetry. *P = 1.5 × 10^−8^ versus WT, **P = 1.0 × 10^−3^ versus WT + Ang II. Insert shows cyclophilin D (CypD) Western blot of endothelial cells isolated from WT and Ec^CypDKO^ mice. (B) Vascular superoxide was measured by DHE and HPLC following accumulation of superoxide-specific product of DHE, 2-hydroxyethidium (insert).^29^ *P = 5.1 × 10^−7^ versus WT, **P = 4.6 × 10^−6^ versus WT + Ang II. (C) Endothelial-dependent relaxation was measured by dose response to acetylcholine (Ach). Aortic relaxation data were analyzed using 2-way analysis of variance (ANOVA) with repeated measurements. Data were analyzed using 2-way ANOVA and Tukey post hoc multiple comparisons. *P = 6.9 × 10^−6^ versus WT, **P = 7.5 × 10^−3^ versus WT + Ang II (*n* = 6). (D) Endothelial nitric oxide (NO) was analyzed by NO spin trap FeDETC_2_ and .^30^ *P = 2.6 × 10^−6^ versus WT, **P = 4.6 × 10^−5^ versus WT + Ang II. Data were analyzed using 2-way ANOVA and Tukey post hoc multiple comparisons. Results are mean ± SD.

To test this hypothesis, we developed tamoxifen-inducible endothelial-specific CypD knockout mice (Ec^CypDKO^). To delete CypD in endothelium, we crossed CypD^lox/lox^ mice (Jackson Labs) with mice transgenic for Cre recombinase driven by a tamoxifen-inducible endothelium VeCad.^22^ The resultant homozygous male CypD^lox/lox^ carrying Cre/VeCAD (Ec^CypDKO^) were studied at 3-5 months of age. Endothelial CypD deletion was induced by injection of tamoxifen (2 mg/30 g of body weight) daily for 5 days. Tissue-specific CypD deletion was confirmed by Western blot analysis of isolated endothelial cells (Figure 1A, insert). Two weeks after tamoxifen injections, mice had undergone telemetry unit placement surgery, and then 2 weeks later received osmotic minipump with Ang II (0.7 mg/kg/day) or saline as a vehicle. Analysis of systolic blood pressure at the end of the 14 days of Ang II infusion in wild-type littermates showed increased systolic blood pressure from 110 mm Hg (sham) to 155 mm Hg (Ang II). Meanwhile, Ang II–induced hypertension in Ec^CypDKO^ mice was significantly attenuated, and systolic blood pressure was changed from 108 mm Hg (sham) to 137 mm Hg (Ang II), which was 18 mm Hg lower than in Ang II–infused wild-type mice (Figure 1A).

To test whether endothelial CypD depletion reduces Ang II–induced vascular oxidative stress, we have measured vascular superoxide in aortas using superoxide probe DHE (50 μm) and performed high-performance liquid chromatography (HPLC) detection of superoxide-specific product, 2-hydroxyethidium.^33^ DHE/HPLC analysis showed a 2-fold increase in vascular superoxide in Ang II–infused wild-type littermates (Figure 1B), which is in line with previously reported data.^34^^,^^35^ Interestingly, depletion of endothelial CypD in Ec^CypDKO^ mice abolished the Ang II–induced superoxide overproduction (Figure 1B). We have tested whether reduced vascular oxidative stress in Ec^CypDKO^ mice protects endothelial-dependent vasorelaxation. Indeed, analysis of acetylcholine-mediated aortic relaxation showed impairment of vasorelaxation in wild-type Ang II–infused mice; meanwhile, endothelial-dependent relaxation was completely preserved in Ang II–infused Ec^CypDKO^ mice (Figure 1C).

Endothelial nitric oxide plays an important role in blood pressure regulation, and vascular oxidative stress reduces nitric oxide levels.^36^ Ec^CypDKO^ mice are protected from oxidative stress (Figure 1B); therefore, we tested whether endothelial CypD depletion preserves nitric oxide production. Endothelial nitric oxide was measured by specific spin trap Fe(DETC)_2_ and ESR, as we have previously described.^30^ ESR analysis showed a significant decrease in endothelial nitric oxide by 52% in aortas from Ang II–infused wild-type mice. In contrast, Ang II infusion in Ec^CypDKO^ mice reduced nitric oxide only by 15%, supporting that endothelial CypD depletion protects nitric oxide levels (Figure 1D).

### Endothelial CypD Depletion Attenuates Glycolytic Switch and Protects Mitochondrial Respiration

**Figure 2.**
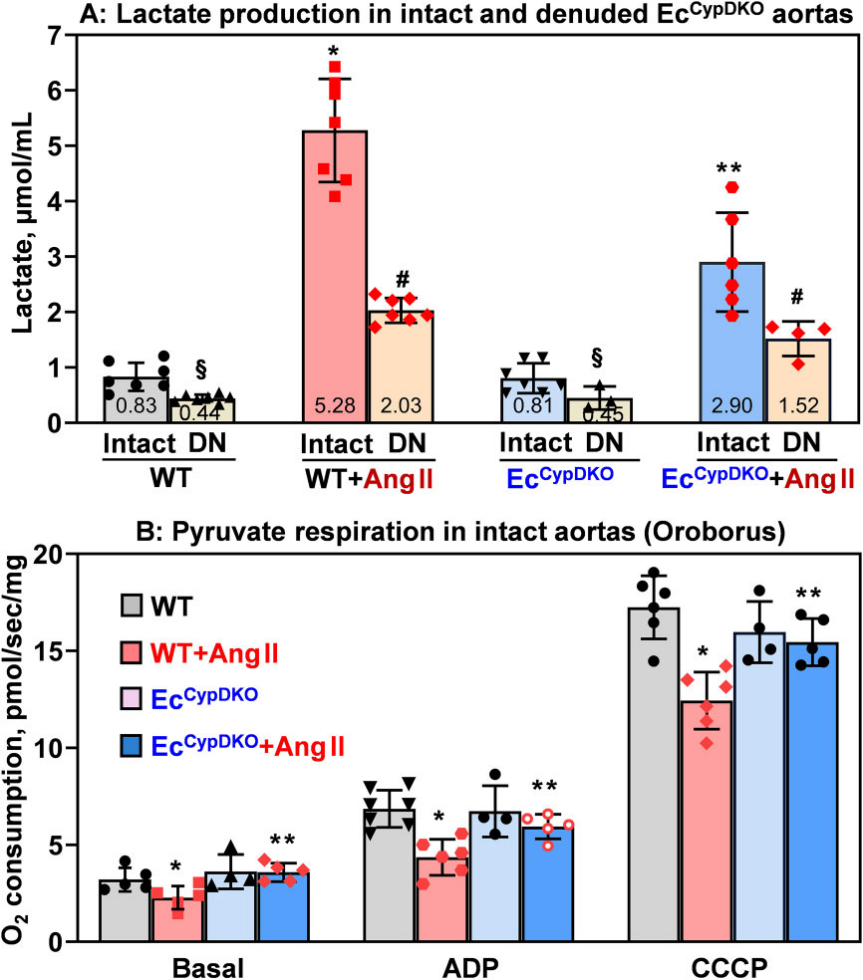
Lactate production in intact and denuded aortas and mitochondrial respiration in intact vessels. Aortas were isolated from sham and angiotensin II (Ang II)–infused (0.7 mg/kg/day, 14 days) male Ec^CypDKO^ and wild-type (WT) mice. (A) Isolated intact and denuded (DN) aortas were placed in DMEM organoid culture (24 h) for analysis of lactate. Data were analyzed using 2-way analysis of variance (ANOVA) and Tukey post hoc multiple comparisons. Results are mean ± SD. *P = 3.8 × 10^−9^ versus WT, **4.3 × 10^−4^ versus WT + Ang II, ^#^*P *= 1.3 × 10^−5^ versus WT + Ang II, **^§^***P *= 3.5 × 10^−3^ versus sham. (B) Mitochondrial respiration was measured in isolated aortas, permeabilized with saponin, washed, and then supplied with pyruvate + malate (basal), ADP, and CCCP. Results are mean ± SD. *P < .001 versus WT, **P < .05 versus WT + Ang II.

We have recently reported that hypertension is linked to maladaptive metabolic switch to vascular glycolysis.^17^ We have tested whether endothelial CypD depletion prevents vascular glycolytic switch and protects mitochondrial function. Analysis of lactate production in isolated aortas showed that a 6-fold increase in vascular glycolysis and endothelial glycolysis (“intact” minus “denuded”) was increased by 8-fold in Ang II–infused wild-type mice. Meanwhile, endothelial CypD depletion in Ec^CypDKO^ mice reduced vascular glycolysis by 45% and attenuated endothelial glycolysis by 210% (Figure 2A). These data demonstrate an important role of endothelial CypD in the maladaptive vascular metabolic alterations in hypertension.

Vascular cells have balanced utilization of glycolysis and mitochondrial respiration,^37^ which are normally coupled,^38^ meaning that glycolysis product pyruvate is utilized by mitochondria. Oxidative stress increases pyruvate dehydrogenase kinase activity inhibiting pyruvate dehydrogenase, which can be a major metabolic step in vascular dysfunction.^18^ We have shown that endothelial CypD depletion prevents vascular oxidative stress (Figure 1B); therefore, we tested whether vascular mitochondrial respiration is preserved in Ang II–infused Ec^CypDKO^ mice. Mitochondrial respiration in saponin-permeabilized aortas from Ang II–infused wild-type mice showed impairment of pyruvate-mediated respiration (Figure 2B). Interestingly, pyruvate-mediated respiration in aortas from Ang II–infused Ec^CypDKO^ mice was not altered compared with sham Ec^CypDKO^ and wild-type mice (Figure 2B).

### Depletion of Endothelial CypD Attenuates Ang II–induced Vascular Hyperpermeability

**Figure 3.**
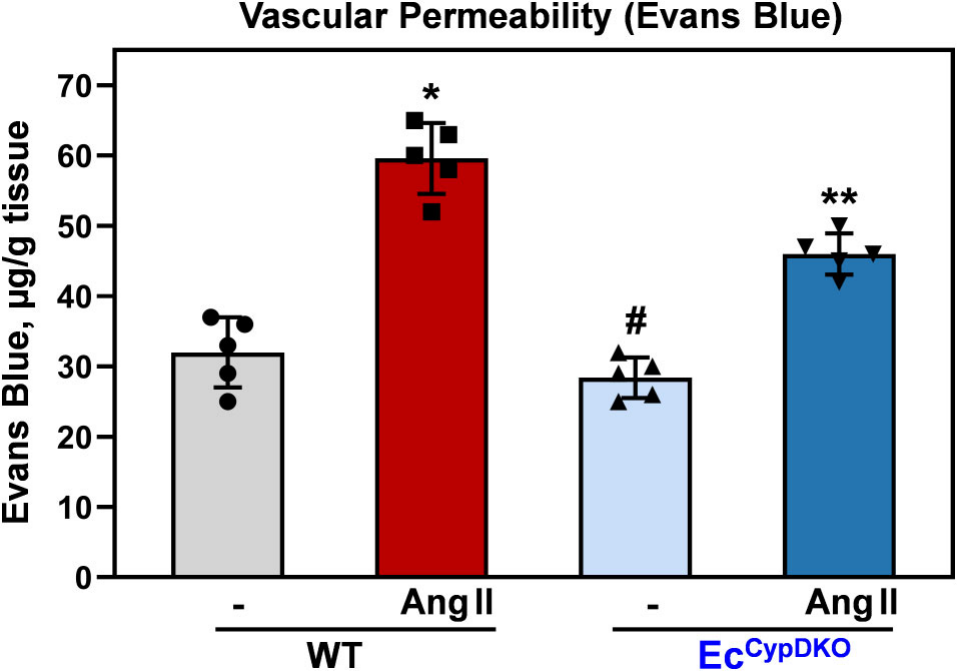
Analysis of vascular permeability in sham and angiotensin II (Ang II)–infused wild-type (WT) and Ec^CypDKO^ male mice. Mice were infused with Ang II (0.7 mg/kg/day) for 14 days, and then vascular permeability was measured by Evans Blue dye in aortas as previously described.^41^ Data were analyzed using 2-way analysis of variance (ANOVA) and Tukey post hoc multiple comparisons. Results are mean ± SD. *P = 2.8 × 10^−6^ versus WT, **P = 2.3 × 10^−3^ versus WT + Ang II, ^#^*P = *2.5 × 10^−4^ versus Ec^CypDKO ^+ Ang II.

It has previously been shown that glycolysis in endothelial cells promotes VE-cadherin endocytosis and proteolytic cleavage,^39^ leading to disruption of VE-cadherin cell-cell junctions, dysfunction of the endothelial barrier, and increased vascular permeability.^40^ We have shown that Ang II–induced endothelial glycolysis is attenuated in Ec^CypDKO^ mice (Figure 2A); thus, we tested whether endothelial CypD depletion protects endothelial barrier function and prevents hypertensive vascular hyperpermeability. It was found that basal vascular permeability in sham Ec^CypDKO^ mice was similar to wild-type littermates as measured by Miles assay and Evans Blue dye.^41^ Angiotensin II–infused wild-type mice increased vascular permeability by 2-fold; however, endothelial CypD depletion in Ec^CypDKO^ mice significantly attenuated Ang II–induced vascular hyperpermeability (Figure 3). These data are in line with previous reports that mitochondrial dysfunction reduces endothelial barrier integrity and increases vascular permeability.^34^ This can increase the access of cytokines and vasoactive substances to the tissue contributing to inflammation, hypertrophy, and end-organ damage, which could be potentially attenuated by CypD depletion.

### Depletion of Smooth Muscle CypD Attenuates Ang II–induced Vascular Dysfunction

**Figure 4.**
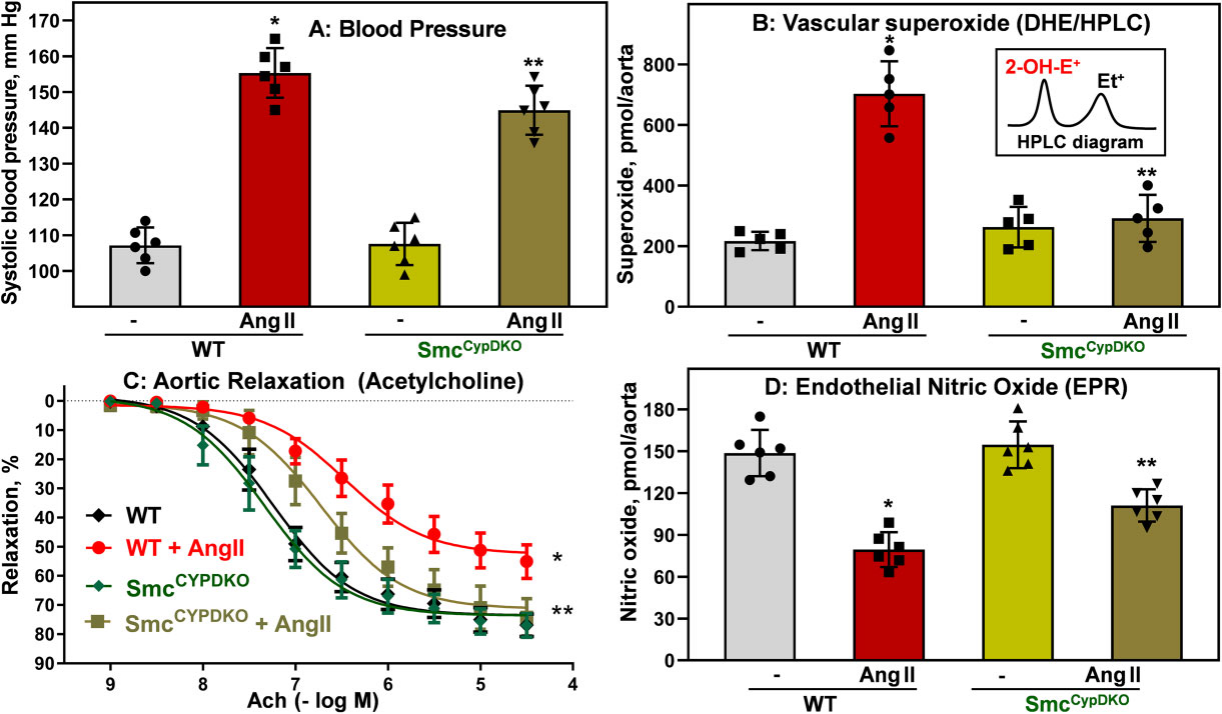
Systolic blood pressure, vascular oxidative stress, nitric oxide (NO), and vasorelaxation in sham and angiotensin II (Ang II)–infused Smc^CypDKO^ and wild-type (WT) male mice. (A) Systolic blood pressure after 14 days of angiotensin II infusion was measured by telemetry. *P = 2.7 × 10^−9^ versus WT, **P = 0.037 versus WT + Ang II. (B) Vascular superoxide was measured by DHE and HPLC following accumulation of superoxide-specific product of DHE, 2-hydroxyethidium (insert).^29^ *P = 4.2 × 10^−6^ versus WT, **P = 2.3 × 10^−5^ versus WT + Ang II. (C) Endothelial-dependent relaxation was measured by dose response to acetylcholine (Ach). Aortic relaxation data were analyzed using 2-way analysis of variance (ANOVA) with repeated measurements. *P = 6.9 × 10^−6^ versus WT, **P = 0.023 versus WT + Ang II (*n* = 6). (D) Endothelial nitric oxide (NO) was analyzed by NO spin trap FeDETC_2_ and electron paramagnetic resonance (EPR).^30^ *P = 2.3 × 10^−6^ versus WT, **P = 0.007 versus WT + Ang II. Data were analyzed using 2-way ANOVA and Tukey post hoc multiple comparisons. Results are mean ± SD.

To test this hypothesis, we have investigated sham and Ang II–induced Smc^CypDKO^ and wild-type mice. To delete CypD in vascular smooth muscle cells, we crossed CypD^lox/lox^ mice (Jackson Labs) with mice transgenic for Cre recombinase driven by tamoxifen-inducible Cre in the vascular smooth muscle (inducible SMMHC Cre) provided by Prof. Stefan Offermanns (University of Heidelberg, Germany).^23^ The resultant homozygous male CypD^lox/lox^ mice carrying Cre/SMMHC (Smc^CypDKO^) were injected with tamoxifen (2 mg/30 g of body weight, daily for 5 days) at 3 months of age to induce smooth muscle CypD deletion. Tissue-specific CypD deletion was confirmed by Western blot. Two weeks after tamoxifen injections, mice had undergone telemetry unit placement, and 2 weeks later received osmotic minipump with Ang II (0.7 mg/kg/day) or saline as a vehicle. Analysis of systolic blood pressure at the end of the 14 days of Ang II infusion in wild-type littermates showed increased systolic blood pressure from 107 mm Hg (sham) to 155 mm Hg (Ang II). Ang II–induced hypertension in Smc^CypDKO^ mice was partially attenuated, and systolic blood pressure was changed from 108 mm Hg (sham) to 145 mm Hg (Ang II), which was 10 mm Hg lower than in Ang II–infused wild-type mice (Figure 4A).

**Figure 5.**
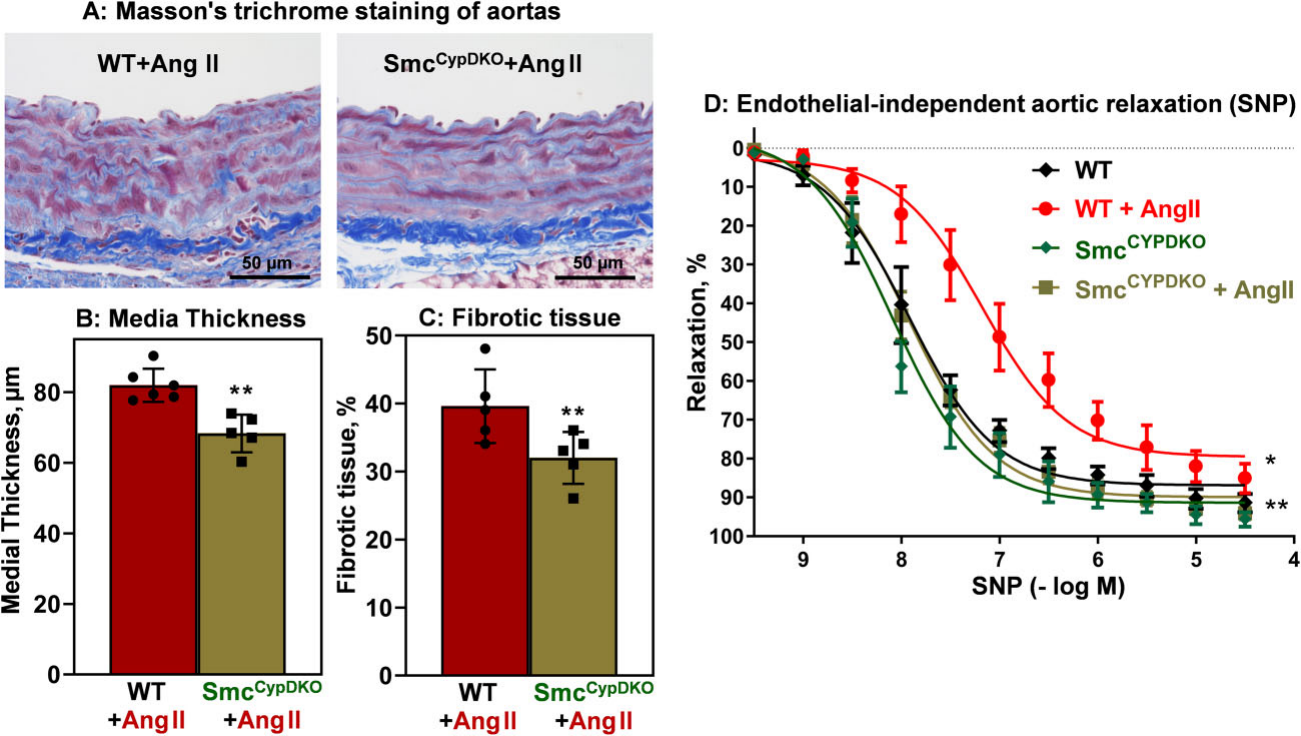
Histopathological analysis of aortas isolated from angiotensin II (Ang II)–infused wild-type (WT) and Smc^CypDKO^ male mice. (A) Representative images of Masson’s trichrome-stained aortic sections. (B) Aortic thickness (**P = .02 versus WT + Ang II) and (C) fibrotic tissue within medial area (**P = .04 versus WT + Ang II) were quantified by Masson’s trichrome and elastic Van Gieson staining.^34^ Data were analyzed using 2-way analysis of variance (ANOVA) and Tukey post hoc multiple comparisons. (D) Endothelial-independent relaxation was measured by dose response to NO donor sodium nitroprusside (SNP). Aortic relaxation data were analyzed using 2-way ANOVA with repeated measurements. *P = .003 versus WT, **P = .005 versus WT + Ang II. Results are mean ± SD (*n* = 6).

It has been reported that global CypD depletion prevents Ang II–induced superoxide overproduction.^16^ In this work we tested whether smooth muscle–specific CypD deletion attenuates vascular oxidative stress. After 14 days of Ang II/sham treatment, mice were euthanized and aortas were isolated for analysis of vascular superoxide using superoxide probe DHE and HPLC.^33^ Ang II infusion in wild-type mice increased vascular superoxide levels by several fold, but depletion of smooth muscle CypD in Smc^CypDKO^ mice prevents Ang II–induced superoxide overproduction (Figure 4B). We have tested whether reduced vascular oxidative stress in Smc^CypDKO^ mice protects nitric oxide from inactivation and attenuates impairment of endothelial-dependent vasorelaxation. Indeed, Ang II–infused hypertension in wild-type mice was associated with a 2-fold decrease in vascular nitric oxide and impaired endothelial-dependent vasorelaxation (Figure 4C and D), while depletion of smooth muscle CypD in Smc^CypDKO^ mice partially protects nitric oxide levels and acetylcholine-induced relaxation (Figure 4C and D; logEC_50_ [Smc^CypDKO^] = 7.3 and logEC_50_ [Smc^CypDKO ^+ Ang II] = 6.7). Endothelial-independent relaxation was reduced in Ang II–infused wild-type mice, but it was protected in Smc^CypDKO ^+ Ang II aortas (Figure 5D). These data support the pathogenic role of CypD in smooth muscle cell oxidative stress in hypertension.

It has previously been shown that vascular oxidative stress is associated with smooth muscle cell hypertrophy and vascular fibrosis.^25^^,^^34^^,^^42^ We have tested whether reduced vascular oxidative stress in Smc^CypDKO^ mice attenuates hypertensive vascular remodeling. Histopathological analysis showed that Ang II infusion significantly increases aortic medial thickness and vascular fibrosis (Figure 5A).^29^ Meanwhile, smooth muscle CypD depletion attenuates Ang II–induced vascular hypertrophy (medial thickness) and reduces vascular fibrosis (Figure 5B and C). These data support the pathogenic role of CypD in hypertensive vascular remodeling.

### Smooth Muscle Cell CypD Depletion Attenuates Ang II–induced Vascular Glycolysis

**Figure 6.**
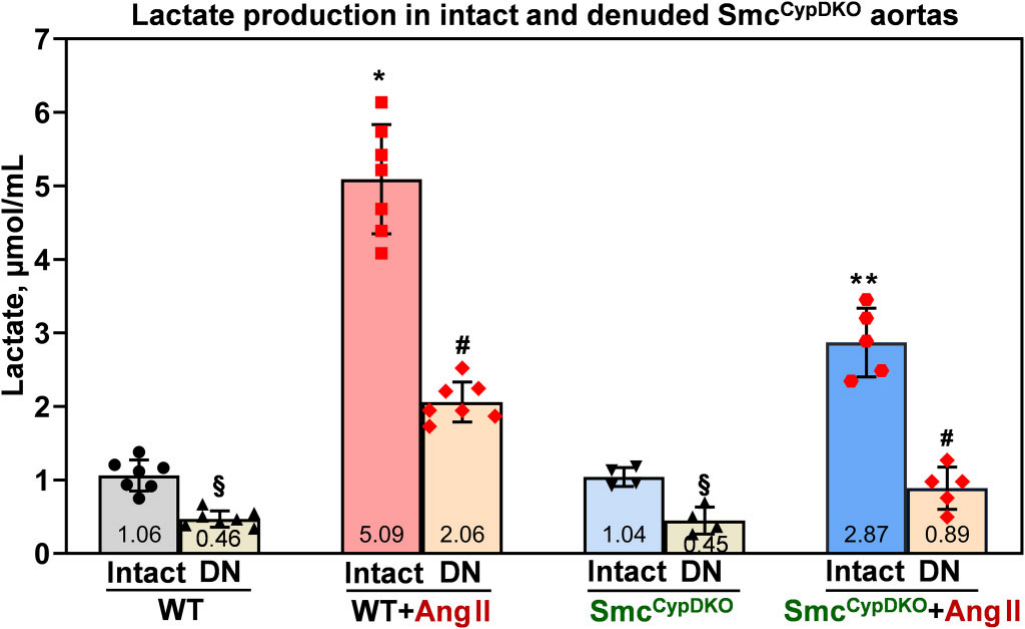
Lactate production in intact and denuded aortas isolated from sham and angiotensin II–infused male Smc^CypDKO^ and wild-type mice. Isolated intact and denuded aortas (DN) were placed in DMEM organoid culture for analysis of lactate. Data were analyzed using 2-way analysis of variance (ANOVA) and Tukey post hoc multiple comparisons. *P = 3.8 × 10^−9^ versus WT intact, **P = 4.2 × 10^−5^ versus WT + Ang II intact, ^#^*P *= 1.1 × 10^−5^ versus Ang II intact, **^§^***P *= 0.05 versus sham intact. Results are mean ± SD.

Pathophysiological role of glycolytic switch in vascular smooth muscle cells promotes aortic aneurysms and vascular fibrosis,^43^^,^^44^ and targeting of vascular glycolysis was proposed to reduce the aneurysmal formation and diminish mortality due to reduced aortic ruptures.^45^^,^^46^ We have tested whether depletion of smooth muscle CypD reduces maladaptive switch to vascular glycolysis by lactate production in intact and denuded aortas isolated from sham and Ang II–induced Smc^CypDKO^ and wild-type mice. Ang II infusion in wild-type littermates increased glycolysis in intact aortas by 5-fold and Ang II infusion in Smc^CypDKO^ mice increased vascular glycolysis only by 2.8-fold (Figure 6). Smooth muscle cell contribution in vascular glycolysis was measured in denuded aortas. Vascular glycolysis in denuded aortas was increased by 4.5-fold in Ang II–infused wild-type mice, and only a 2-fold increase was detected in Ang II–infused Smc^CypDKO^ mice. These data demonstrate an important role of smooth muscle CypD in the maladaptive vascular glycolytic switch in hypertension.

## Discussion

This study provides the first evidence that cell-specific CypD depletion in endothelial and smooth muscle cells attenuates vascular dysfunction and hypertension. Animal studies in the Ang II–induced model of hypertension showed that cell-specific CypD knockout in endothelial cells prevents vascular oxidative stress, preserves endothelial-dependent relaxation, protects endothelial nitric oxide production, and attenuates Ang II–induced vascular hyperpermeability, indicating that endothelial CypD depletion protects the endothelial function (Figures 1 and 3). As expected, endothelial CypD depletion protects vascular mitochondrial respiration and significantly reduces maladaptive switch of vascular metabolism to glycolysis (Figure 2). Protection of endothelial function and metabolism in Ec^CypDKO^ mice was coupled with diminished Ang II–induced hypertension (Figure 1). Studies of Smc^CypDKO^ mice showed that smooth muscle CypD depletion inhibits vascular superoxide overproduction, reduces inactivation of endothelial nitric oxide, diminishes impairment of endothelial-dependent relaxation, and attenuates Ang II–induced vascular hypertrophy and fibrosis (Figures 4 and 5). It was found that smooth muscle CypD depletion diminished Ang II–induced vascular metabolic switch to glycolysis (Figure 6). Protection of smooth muscle cell metabolism and function in Smc^CypDKO^ mice was associated with partial attenuation of Ang II–induced hypertension (Figure 4). These data support the pathogenic role of mitochondrial CypD in vascular dysfunction and hypertension.

It has previously been suggested that CypD can act as a master regulator of mitochondrial function.^9^ Indeed, CypD acts as a scaffold protein by binding to multiple mitochondrial proteins.^47^ It regulates coupling of the electron transport chain and ATP synthesis.^9^ Constitutive global CypD depletion in *CypD^−/−^* mice showed acetylation of fatty acid oxidation proteins associated with inhibition of fatty acid oxidation in the cardiac mitochondria isolated from *CypD^−/−^* mice.^48^ This suggests a novel link between CypD and mitochondrial acetylation,^49^ and it was proposed that CypD modulates mitochondrial acetylome.^48^ This creates a potential paradox since *CypD^−/−^* mice are protected against ischemic injury, but cardiac CypD depletion can alter metabolism and promote heart failure.^50^ On one hand, CypD can induce mitochondrial permeability pore opening, mitochondrial swelling, and cell death.^51^ On the other hand, CypD supports mitochondrial homeostasis.^50^ We think there are several explanations for this CypD paradox. First, constitutive CypD deletion may lead to the developmental problems and cause the adaptive epigenetic and metabolic changes in *CypD^−/−^* mice. Second, our clinical studies and basic medical research did not show significant alterations in CypD expression; however, CypD S-glutathionylation^16^ and CypD-acetylation^17^ are increased in vascular dysfunction and hypertension, suggesting a pathogenic role of CypD post-translational modifications, which can lead to switch from homeostatic to pathogenic “gain of function.” This raises the question whether conditional blocking of CypD is detrimental. Our data did not show any deleterious effects of conditional CypD depletion in tamoxifen inducible in Ec^CypDKO^ and Smc^CypDKO^ mice. Inducible CypD depletion in endothelial and smooth muscle cells did not affect the basal metabolism, endothelial nitric oxide, vasorelaxation, blood pressure, and basal heart rate (Supplemental Figure 1S). Mitochondria have tremendous metabolic plasticity, and our data suggest that CypD is dispensable in adult homeostatic functions. We suggest that conditional CypD depletion or pharmacological inhibition of CypD can be effectively compensated by other mitochondrial pathways. Meanwhile, blocking CypD can be beneficial to prevent CypD switch from homeostatic to pathogenic “gain of function” in pathological conditions.

In this work, we studied tamoxifen-inducible Ec^CypDKO^ and Smc^CypDKO^ male mice using the Ang II model of hypertension. There are several limitations of this experimental approach. First, our study was limited by the analysis of the male mice and did not include the female mice. Unfortunately, breeding transgenic mice with inducible SMMHC Cre located in the Y chromosome provides only Smc^CypDKO^ male mice.^23^ A new smooth muscle tamoxifen-inducible Cre mice with Myh11-driven smooth muscle cells expression integrated in chromosome 2 (Jackson Laboratory, strain # 037658) allows studies in both male and female mice.^52^ Previous animal studies showed attenuated Ang II–induced hypertension in female mice compared with male littermates.^53^ This makes it difficult to study the protective effect of CypD depletion in females, which have already significant protection. We did not notice sex differences in CypD expression.^17^ Previous studies did not reveal sex differences in body weight, lifespan, and behavioral activity in response to CypD depletion.^54^ Other studies found that global CypD deletion can impair fatty acid β-oxidation and stimulate glucose metabolism.^55^ Women utilize fatty acids more as a primary energy source, while men rely more on carbohydrates for energy,^56^ and mitochondria from females have lower oxidative stress than males^57^ suggesting a potential sex difference in CypD function. Proteomic studies showed very little difference between males and females in mitochondrial antioxidant proteins and fatty acid oxidation enzymes,^58^ which may suggest a potential role for the post-translational modifications in sex-specific mitochondrial functions. We suggest that future studies must elucidate the potential sex differences in the pathogenic CypD post-translational modifications such as cysteine S-glutathionylation and lysine acetylation.^16^^,^^17^

The second limitation of this study is dealing with the use of the Ang II model. Future studies must be directed to test the CypD function in volume-mediated DOCA-salt hypertension and salt-induced hypertension. The third limitation of this study deals with the focus on aortic physiology, which does not reflect the microvascular function. Meanwhile, resistant vessels such as arteries and microvasculature define the vascular peripheral resistance and, therefore, arterial pressure in hypertension. Previous studies showed impaired endothelial-dependent relaxation both in microvasculature,^59^ resistant arteries and conduit vessels such as aorta (see Supplemental Figure 2S). We have recently shown that protection from mitochondrial oxidative stress preserves endothelial-dependent relaxation in mesenteric arteries in the Ang II model of hypertension.^35^ Meanwhile, the recovery of endothelial function may vary due to distinct metabolic conditions of these vessels^60^ and differences in the regulation of endothelial-dependent dilation.^61^ Future studies are needed to define the role of CypD in the pathophysiological alterations of relaxation/contraction of resistant vessels and microvasculature. Fourth, our studies were limited to 3- to 5-month-old mice having CypD depletion for 2 months only. Future studies must test the potential off-target and systemic effects of long-term CypD depletion or CypD inhibition in adult and aged animals. Other limitations include the weakness of the data in respiration studies (Figure 2) due to a low number of samples in denuded aortas groups, the lack of blood pressure tracing for the entire period of the study, and the lack of whole vessel images in Ec^CypDKO^ and Smc^CypDKO^ studies (Figure 5). Future studies should include more than 6 samples per group in metabolic studies, analysis of blood pressure during the entire study period, and more thorough histopathological analysis.

Endothelial-smooth muscle cell interaction is important in vascular homeostasis, blood vessel tone, and blood pressure regulation.^62^^,^^63^ This is mediated by direct cell-to-cell contact and through paracrine signaling. Our data showed an intriguing “metabolic” crosstalk between endothelial and smooth muscle cells. We found that depletion of endothelial CypD reduces Ang II–induced endothelial glycolysis (intact aorta minus denuded aorta) by 2.1-fold and diminishes smooth muscle cell glycolysis (denuded aortas) by 1.34-fold. Interestingly, depletion of smooth muscle CypD was also not limited to the effect on smooth muscle glycolysis (2.3-fold), but it also reduced endothelial cell glycolysis (1.53-fold). It is not clear whether this is a direct metabolic interaction or a result of redox crosstalk. Oxidative stress in smooth muscle cells promotes redox alterations in endothelial cells,^64^ and vice versa endothelial oxidative stress promotes redox-dependent smooth muscle cell activation, differentiation, and hypertrophy.^36^ Vascular oxidative stress was inhibited in both Ec^CypDKO^ and Smc^CypDKO^ mice; therefore, cell-specific CypD depletion had the “global” antioxidant effect on the entire vasculature. This antioxidant effect of CypD depletion can attenuate the Ang II–induced glycolysis not only in specific cells but in the entire vasculature due to direct redox regulation of HIF-1α and glycolysis.^65^ Meanwhile, recent studies showed that increased lactate can directly affect vascular cell functions, and diminished lactate production in endothelial or smooth muscle cells can substantially reduce total vascular levels of lactate and, therefore, attenuate the lactate-mediated vascular alterations.^39^^,^^40^ Interestingly, males exhibit higher glycolytic activity in blood vessels compared with females,^66^ which is in line with higher mitochondrial activity,^67^ lower oxidative stress,^68^ and renin–angiotensin–aldosterone system^69^^,^^70^ in females compared with males. Meanwhile, the specific role of mitochondria and CypD in these redox and metabolic vascular alterations needs further investigation.

Despite significant progress in mitochondrial studies, there are many critical gaps in knowledge, which hinder clinical translation of targeting CypD. In this work, we used a preventive model and showed that endothelial and smooth muscle cell CypD depletion significantly prevents Ang II–induced vascular dysfunction. Meanwhile, we do not know whether CypD depletion or CypD inhibition after onset of hypertension is beneficial. We may anticipate that blocking CypD can stop disease progression; however, it is not clear whether this would rescue or improve vascular function. We have described that CypD depletion or CypD deacetylation attenuates endothelial dysfunction and hypertension^16^^,^^17^; however, we do not know the specific molecular mechanisms of CypD activation. It is conceivable that targeting “activated” CypD can be more beneficial rather than blocking all CypD in the whole body. Preventive studies using CypD depletion do not allow us to define the causative role of specific pathogenic mechanisms. Development of animal models with “activated” CypD can provide invaluable information regarding specific mitochondrial and cellular pathogenic pathways. This would allow to define the causative role of specific CypD post-translational modifications and help us with the development of pharmacological approaches to specific targeting of “activated” CypD.

Nonimmunosuppressive CypD blockers based on cyclosporine derivatives were developed by Novartis^71^ and NeuroVive Pharmaceutical, which are promising in cardiovascular clinical studies.^72^ Clinical translation of these drugs was hindered by off-target effects, potentially, due to inhibition of CypD peptidyl-prolyl cis-trans isomerase activity.^9^ CypD acetylation leads to a “gain of function”^73^ promoting mPTP opening, and specific targeting of acetylated CypD may be beneficial in cardiovascular disease. It is possible that specific binding to the CypD acetylation site can block the detrimental CypD functions without an off-target effect on the CypD homeostatic role. This can provide an important basis for the development of new nonimmunosuppressive CypD inhibitors such as NV556,^71^ which can be promising for future clinical studies.^72^

Mitochondrial dysfunction is associated with hypertension and cardiovascular conditions; however, the specific molecular mechanisms of mitochondrial dysfunction and their causative role are not clear. Understanding these molecular mechanisms is important for the development of new therapies. Mitochondria are critical in cellular metabolism and function, and a new concept suggests that mitochondria are a common pathobiological target for multiple cardiovascular risk factors.^4^ Multiple pathophysiological pathways, therefore, cooperatively induce mitochondrial dysfunction leading to metabolic alterations and oxidative stress, driving vascular epigenetic and phenotypic dysregulation. In this respect, alterations of “master” regulators of mitochondrial function such as CypD can provide both mechanistic insight and novel therapeutic targets. We suggest that strategies directed to block pathogenic CypD pathways may have therapeutic potential in vascular dysfunction, hypertension, and hypertensive end-organ damage.

## Supplementary Material

zqaf006_Supplemental_File

## Data Availability

The data underlying this article will be shared on reasonable request to the corresponding author.
