# Supplementary material for: Depletion of Mitochondrial Cyclophilin D in Endothelial and Smooth Muscle Cells Attenuates Vascular Dysfunction and Hypertension
Source: Function (Oxf). 2025 Feb 7;6(2):zqaf006. doi: 10.1093/function/zqaf006 (PMC11931617; doi:10.1093/function/zqaf006)
Supplement: zqaf006_Supplemental_File [file zqaf006_supplemental_file.pdf]

SUPPLEMENTAL MATERIAL

Depletion of mitochondrial CypD in endothelial and smooth muscle cells attenuates vascular dysfunction and hypertension

Function 2025

Anna Dikalova,[1] Mingfang Ao,[1] Louise Lantier,[2] Sergey Gutor,[3] and Sergey Dikalov [1§]

[1] Vanderbilt University Medical Center, Nashville, Tennessee, USA

[2] Vanderbilt University, Nashville, Tennessee, USA

[3] University of Michigan; Ann Arbor, Michigan, USA

[§] To whom correspondence should be addressed:

Sergey Dikalov, Ph.D.

Division of Clinical Pharmacology

Vanderbilt University Medical Center

2220 Pierce Ave, PRB 554

Nashville, TN 37232

Tel.: 615-936-3694

E-mail: sergey.dikalov@vumc.org

Supplemental Figures

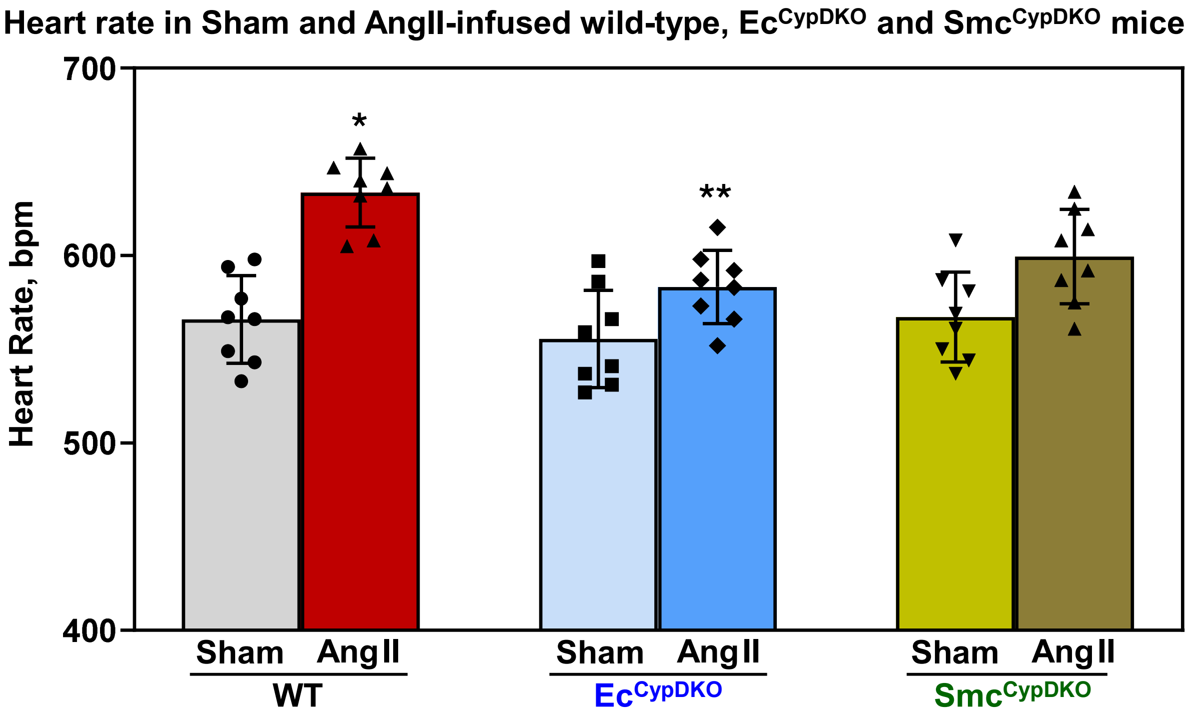

Supplemental Figure 1S: Heart rate in Sham and angiotensin II-infused wild-type, Ec^{CypDKO} and Smc^{CypDKO} male mice. Mice were implanted with osmotic pumps containing angiotensin II (0.7 mg/kg/day) or saline as a vehicle. The data shows heart rate after 14-days of angiotensin II infusion. Results are mean ± STD. **P*=5.3×10^{-5} vs WT Sham. **P*=0.013×10^{-5} vs WT+AngII.

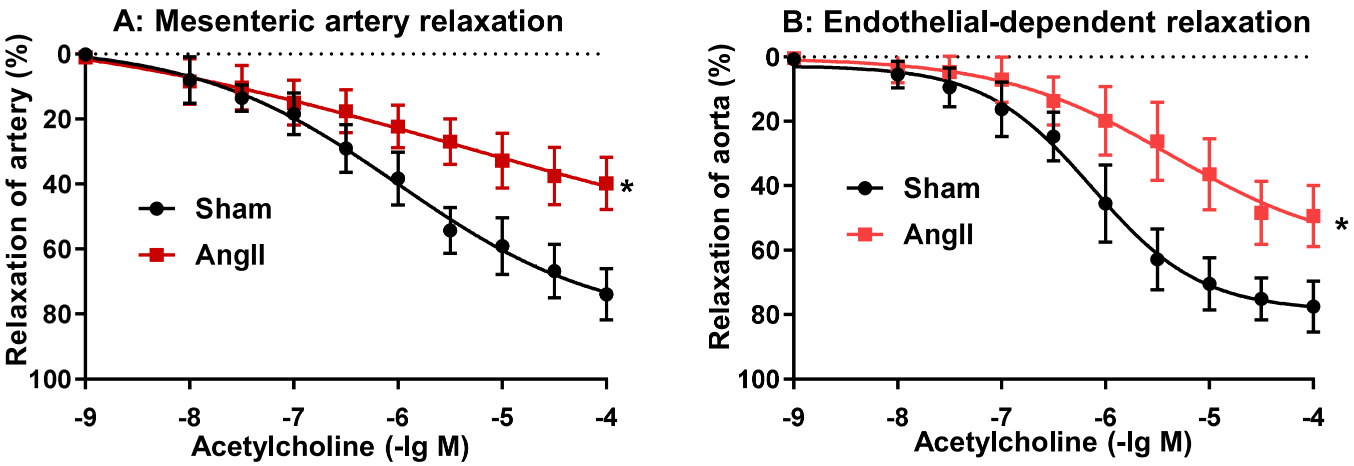

Supplemental Figure 2S: Endothelial-dependent relaxation of mesenteric arteries (A) and aortas (B) in Sham and angiotensin II-infused wild-type male mice (0.7 mg/kg/day, 14-days). Results are mean ± STD. **P*<0.001 vs Sham (n=6).
